# Outcome in serous ovarian cancer is not associated with LATS expression

**DOI:** 10.1007/s00432-019-03037-4

**Published:** 2019-10-04

**Authors:** Céline Montavon, Gregor R. Stricker, Andreas Schoetzau, Viola Heinzelmann-Schwarz, Francis Jacob, André Fedier

**Affiliations:** 1 Gynecological Cancer Center, Hospital for Women, University Hospital Basel and University of Basel, 4021 Basel, Switzerland; 2 Ovarian Cancer Research, Department of Biomedicine, University Hospital Basel and University of Basel, 4031 Basel, Switzerland; 3 Glyco-Ooncology, Ovarian Cancer Research, Department of Biomedicine, University Hospital Basel and University of Basel, 4031 Basel, Switzerland

**Keywords:** Ovarian cancer, Large tumor suppressor (LATS), Outcome, Epithelial–mesenchymal transition (EMT), Drug sensitivity, Transcriptomics

## Abstract

**Background:**

Large tumor suppressor (LATS) proteins are putative tumor suppressors and poorly expressed associated with poor outcome in many cancers. A recent immunohistochemistry study showed that LATS protein expression correlated with poor outcome in serous ovarian cancer.

**Materials and methods:**

We analyzed *LATS* expression in various ovarian cancer transcriptomic data sets and immunohistochemically assessed LATS protein expression in a Swiss ovarian tumor cohort. Results were compared to clinicopathological characteristics and outcome. We also compared LATS protein expression in serous ovarian cancer cell lines to their EMT status (Western blotting) and drug sensitivity (MTT assay).

**Results:**

The analysis of 15 different transcriptomic data sets showed that *LATS2* was associated with poorer outcome, while *LATS1* was irrelevant (HR = 1.19 and HR = 1.00, respectively). The TCGA-RNASeqV2 data set showed that low *LATS1* and *LATS2* were associated with better survival in serous ovarian carcinoma. Despite heterogeneity among the different data sets, *LATS* expression is not an indicator of survival in serous ovarian cancer and *LATS2* expression may even be tumorigenic. LATS expression was neither associated with survival nor with the stage and grade in the Swiss cohort. It was low in cystadenoma, intermediate in carcinoma, and high in borderline tumors and was higher in serous than mucinous ovarian carcinoma. LATS protein expression extent was comparable in epithelial-, intermediate-, and mesenchymal-type ovarian cancer cells and was not associated with drug sensitivity.

**Conclusion:**

These results are largely incompatible with a tumor-suppressive function of LATS in ovarian cancer, and LATS protein level is also not an indicator for drug sensitivity and EMT status of ovarian cancer cells.

## Introduction

Large tumor suppressor (LATS) family proteins LATS1 and LATS2 have been proposed to be tumor suppressors. They have been reported to govern cellular homeostasis by preventing cell proliferation and migration, by inducing cell death and senescence, and by regulating cell cycle checkpoints to maintain genetic stability (Visser and Yang 2010; Furth and Aylon 2017). Consistent with their proposed tumor-suppressive function, LATS proteins have been reported to be down-regulated in various cell types including breast cancer (Morinaga et al. 2000), non-small lung cancer (Lin et al. 2014), and gastric cancer (Son et al. 2017). However, LATS was reported to be overexpressed in nasopharyngeal cancer (Zhang et al. 2010), suggesting that the function of LATS remains controversial and may be tissue- and cancer-type dependent.

Large tumor suppressor1 and LATS2 are the core kinases of the Hippo pathway, in which LATS1 and LATS2 become activated by upstream kinases in response to stimuli and then phosphorylate and thereby repress the nuclear activity of two transcriptional co-factors YAP and TAZ by their cytoplasmic retention, eventually preventing the transcription of tumor-promoting genes. The Hippo signaling pathway plays a critical role in organogenesis, tumorigenesis, metastasis, stem cell differentiation and renewal, and mechanotransduction (Furth and Aylon 2017; Meng et al. 2016; Janse van Rensburg and Yang 2016; Yu et al. 2015). Accordingly, YAP/TAZ are activated, whereas LATS1/2 are inactivated in many human malignant tumors (Plouffe et al. 2015; Zanconato et al. 2016).

Clinically, LATS expression can have prognostic value. LATS1 and LATS2 expressions have been reported as significant markers for good prognosis in patients with gastric cancer (Son et al. 2017), reduced LATS1 correlated with poor outcome with breast cancer patients (Takahashi et al. 2005), and reduced LATS2 correlated with poor survival in acute lymphoblastic leukemia (Jiménez-Velasco et al. 2005).

The role of LATS in ovarian cancer remains poorly understood. Epithelial ovarian cancer is the leading cause of death in gynecologic cancer. In patients with advanced FIGO stage serous ovarian cancer, the most common, aggressive and deadly type, the 5-year survival rate is  < 30%. This poor outcome is due to the lack of early disease-specific symptoms and reliable tools for early diagnosis, as well as ineffective therapy for advanced disease (Ozols 2006; Bowtell et al. 2015). Recently, a study evaluated immunohistochemistry data from Chinese ovarian cancer samples and showed that LATS1 and LATS2 expression was reduced in serous ovarian cancer patients associated with shorter survival and increased recurrence, while LATS1 and LATS2 were highly expressed in fallopian tube and LATS1 was expressed in normal ovarian tissue (Xu et al. 2015).

To validate these previous data, we investigated LATS expression (1) and clinical outcome in ovarian cancer using various publicly accessible transcriptomic data sets and (2) in our Swiss ovarian cancer patient cohort and related the results to various clinicopathological parameters. As LATS expression has also been shown to regulate epithelial-mesenchymal transition (EMT), a cellular program that promotes invasion and metastasis during cancer development, and modulate drug responses (Nozaki et al. 2019; Zhang et al. 2012; Takahashi et al. 2007; Kawahara et al. 2008), we determined LATS protein expression in a panel of ovarian cancer cell lines and investigated (3) whether LATS expression in these cell lines was an indicator for the EMT state of the cells and for the sensitivity to chemotherapeutic drugs.

## Materials and methods

### Transcriptomic database analysis

Transcriptomic ovarian databases were analyzed using the Bioconductor package “curatedOvarianData” which represents a manually curated data collection for gene expression meta-analysis of patients with ovarian cancer (Ganzfried et al. 2013). The results are presented as hazard ratios (*HR*) of *LATS1* and *LATS2* with corresponding 95% CI using forest plots. In addition, TCGA RNASeqV2 data were analyzed in more detail, presenting results from Cox regression using the two quantiles (0.05, 0.95) for *LATS1* and *LATS2.*

### Patient cohort, tissue microarray immunohistochemistry, and ethical approval

The patient cohort was previously recruited at the University Hospital Zurich, Switzerland from 1990 to 2007 and the respective tissue microarray (TMA) was used for the immunohistochemistry (IHC) experiments (described in: Jacob et al. 2012). Deparaffinized and stained sections were incubated with anti-LATS1/2 antibody (GTX87014; Lucerna Chem, Lucerne, Switzerland) as per standard laboratory and manufacturer’s protocols. This antibody recognizes endogenous levels of total LATS 1/2 protein. Tissue slides were counterstained with hematoxylin. Sections were dehydrated and coverslipped. Immunostaining was scored by the weighted average score (intensity: 0–3, coloring: 0–100% of LATS1/2 expression) by three trained scientists (CM, GS, VHS) independently and discrepancies were resolved by consensus. The cohort consisted of 710 samples on three TMAs from initially 271 patients. There were 200 samples with incomplete data or with non-evaluable scores. 510 samples had evaluable scores, whereof 122 had no LATS expression and 388 had LATS expression. For patients with more than one sample, the scores were averaged. From the resulting 205 patients, a miscellaneous group of 9 patients was excluded due the low patient numbers and unclear malignancies. The final overall cohort consisted of 196 ovarian tumor patients: 85 (benign) cystadenomas, 32 borderline, and 79 carcinoma (Table 1).

**Table 1:** Overall ovarian tumor cohort description

	All tumors	Cystadenoma	Borderline	Carcinoma	*N*
	*N* = 196	*N* = 85	*N* = 32	*N* = 79	196
Tumor type					196
Cystadenoma	85 (43.4%)	85 (100%)			
Borderline	32 (16.3%)		32 (100%)		
Carcinoma	79 (40.3%)			79 (100%)	
Histological subtype					196
Clear cell	14 (7.2%)		1 (3.12%)	13 (16.5%)	
Endometrioid	12 (6.1%)			12 (15.2%)	
Mucinous	43 (21.9%)	31 (36.5%)	10 (31.2%)	2 (2.53%)	
Serous	127 (64.8%)	54 (63.5%)	21 (65.6%)	52 (65.8%)	
Stage					107
I + II	48 (44.9%)		25 (83.3%)	20 (27.0%)	
III + IV	59 (55.1%)		5 (16.7%)	54 (73.0%)	
Grade					110
1	4 (3.6%)			4 (5.1%)	
2^a^	23 (20.9%)			23 (29.5%)	
3	51 (46.4%)			51 (65.4%)	
Borderline	32 (29.1%)		32 (100%)		
Score^b^	0.25 [0.06;0.63]	0.10 [0.00;0.26]	0.73 [0.42;0.98]	0.38 [0.15;0.66]	196
Medsplit^c^					196
Low expression	100 (51.0%)	63 (74.1%)	5 (15.6%)	32 (40.5%)	
High expression	96 (49.0%)	22 (25.9%)	27 (84.4%)	47 (59.5%)	

*N* number of patients

^a^Includes the G2 serous high-grade carcinomas; ^b^score, median and IQR [interquartile range]; ^c^median split

Ethics approval was obtained from the Swiss Ethical Cantonal Department SPUK (approval #StV06/2006) and the Ethical Committee of Nordwest- und Zentralschweiz, Switzerland (EKNZ 2015 ± 436). Neither written nor oral consent was necessary for this retrospective study and data accession was anonymous. The whole study was performed according to the Declaration of Helsinki and local laws and regulations.

### Cell lines and cell culture

A2780, BG-1, CaOv3, IGROV-1, Kuramocchi, OAW42, OVCAR-3, OVCAR-4, OVCAR-8, SKOV-3, TOV112D and TYK-nu (parental) ovarian cancer cell lines; FT190 and FT194 fallopian tube cell lines; and HOSE 6.3 and HOSE 17.1 human ovary surface epithelial cells were cultured in RPMI (R8758, Sigma-Aldrich, Buchs, Switzerland) supplemented with 10% fetal bovine serum (FBS; Sigma-Aldrich) and penicillin/streptomycin (100 U/mL/100 μg/mL; Sigma-Aldrich) at 37 °C in a 95% humidified atmosphere containing 5% CO_2_. All cell lines were routinely tested for mycoplasma infection. Cisplatin-resistant A2780/CP and TYK-nu(R) cell lines were obtained from the National Cell Bank of Iran (NCBI) and the JCRB Cell Bank Japan, respectively, and these cell lines had acquired cisplatin resistance generated by stepwise incubation of the parental cells with inclining cisplatin concentrations (Masuda et al. 1988; Yoshiya et al. 1989). Paclitaxel-resistant IGROV1-PXL cells were generated in our laboratory by stepwise exposure of parental IGROV-1 cells to increasing concentrations of paclitaxel (Kohler et al. 2017). They were also cultured as described above.

### Western blot analysis

Western blotting was used to determine the protein expression in the cell lines and was performed according to standard laboratory protocols. Briefly, cell lysates were obtained from subconfluent cultures at the time of harvest. Cells were lysed with RIPA buffer (9806, Cell Signaling; BioConcept, Allschwil, Switzerland). Protein concentration was determined by the BCA Protein Assay (23227; Pierce, Perbio Science, Switzerland). Twenty micrograms of protein was loaded and separated using sodium dodecyl sulfate-polyacrylamide gel electrophoresis (SDS-PAGE), followed by blotting onto polyvinylidene difluoride (PVDF) membranes (162-0177, BioRad, Crissier, Switzerland). Proteins were detected with specific primary antibodies and appropriate secondary antibody (HRPO-conjugated anti-mouse (7076, Cell Signaling) or HRPO-conjugated anti-rabbit (7074, Cell Signaling). The primary antibodies were rabbit anti-LATS1 (9153, Cell Signaling), rabbit anti-LATS2 (ab110780, Abcam, Lucerne Chem), rabbit anti-E-cadherin (3195, Cell Signaling), mouse anti-vimentin (MA5-11883, Invitrogen, Thermo Fisher Scientific, Wohlen, Switzerland), and mouse anti-MDR1 (sc-13131, Santa Cruz, Lab Force, Muttenz, Switzerland) antibodies. Rabbit anti-tubulin antibody (2148, Cell Signaling) was used as a sample loading control. Complexes were visualized by enhanced chemiluminescence (Dura West, Pierce, Perbio Science) and autoradiography. Quantitative analysis of the complexes (intensity on autoradiogram) was performed by densitometry (normalized against tubulin) using Image J software.

### MTT assay

The MTT assay was used to determine the sensitivity of the cell lines to chemotherapeutic drugs and was performed as follows: cells were seeded into 96-well plates and treated on the next day with chemotherapeutic drugs for 72 h, followed by the addition of 20 μl of MTT dye (Sigma-Aldrich) in PBS (final concentration: 0.5 mg/ml). Chemotherapeutics were purchased from the following suppliers: carboplatin (Labatec SA, Geneva, Switzerland) and cisplatin, doxorubicin, and paclitaxel (Sigma-Aldrich). After 3 h, the medium was removed and the purple crystals were dissolved in 200 μl DMSO. Optical density (absorbance at 540 nm) was measured with a SynergyH1 Hybrid Reader (Biotek, Zurich, Switzerland). Data (mean ± SD of at least four independent experiments performed in quadruplets) are presented as function of drug concentration. IC50 values were calculated by linear extrapolation.

### Statistical analysis

Descriptive statistics is presented as median (IQR) or counts and percentages as appropriate. To compare weighted average scores between subgroups, Dunn’s test following Kruskal–Wallis tests was performed. To analyze the influence of LATS1 and LATS2 on time-to-event data, the expression values were split by medians providing high/low groups. Kaplan–Meier statistics were presented as graphs and median times with 95% confidence intervals. Time to event curves were compared using Log-rank tests presented as *p* values: a *p* value < 0.05 is considered as statistically significant. All evaluations were done using the statistical software R version 3.5.1 (https://www.R-project.org/). *P* values were considered as exploratory and therefore not adjusted for multiple comparisons. For comparisons regarding drug sensitivity and LATS expression in cell lines, the mean ± SD values were calculated and statistical analysis was performed using the two-tailed Student’s *t* test, where *p* values < 0.05 were considered statistically significant.

## Results

### LATS expression is not associated with better outcome in ovarian cancer (transcriptomic data)

**Fig. 1:**
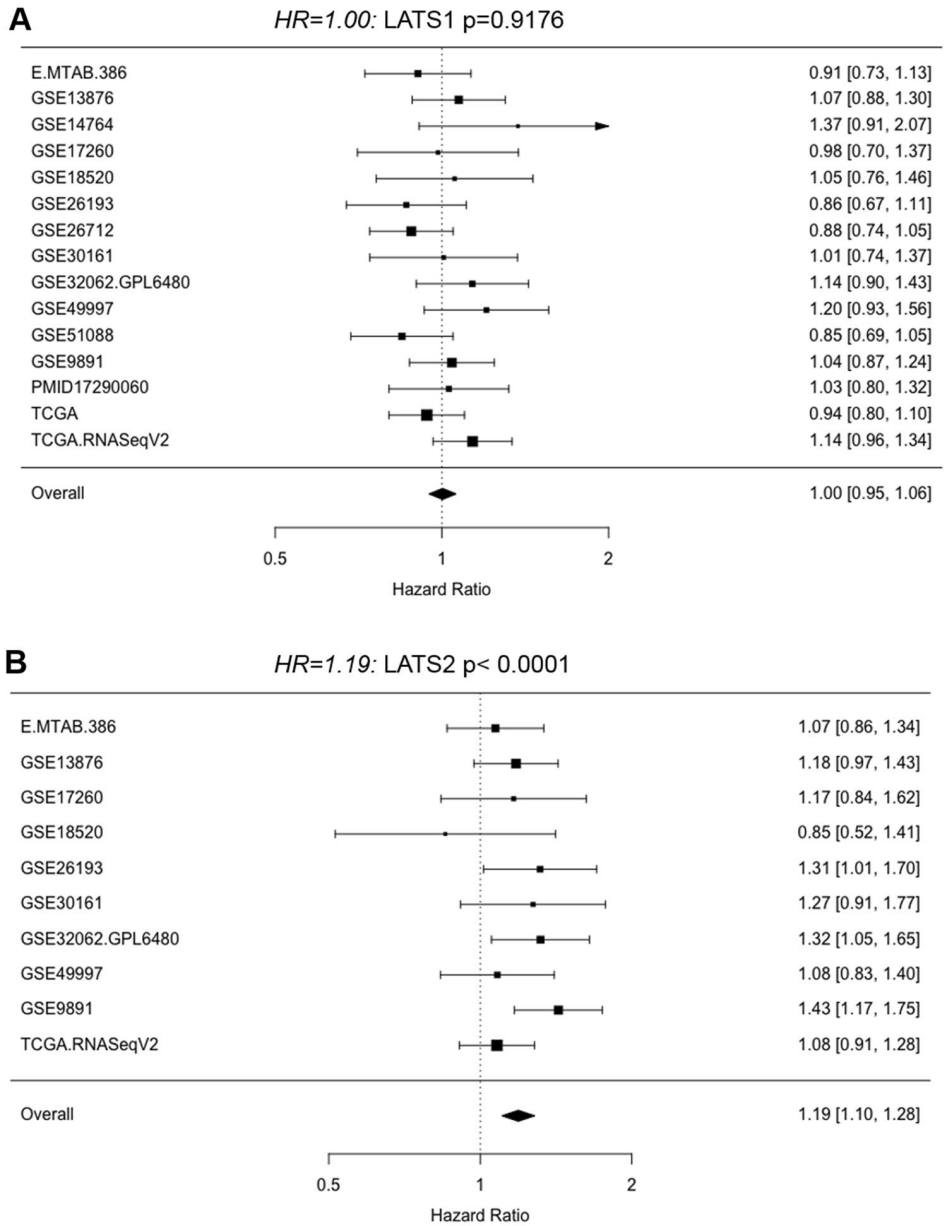
*LATS1* and *LATS2* expression and outcome in ovarian cancer (transcriptomic data). **a**, **b** Forest plot presenting hazard ratios (HR) computed from different transcriptomic data sets for *LATS*1 and *LATS2* in ovarian cancer

**Fig. 2:**
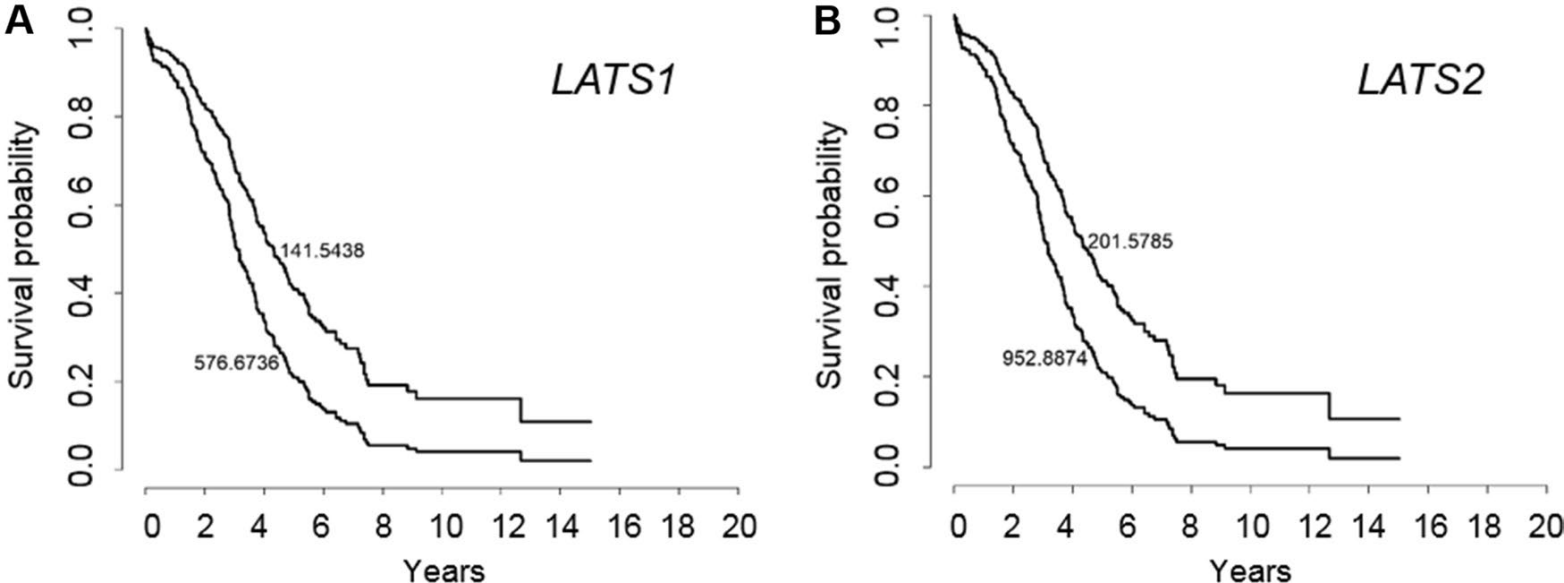
Kaplan–Meier presentation of log(*LATS1*) (**a**) and log(*LATS2*) (**b**) on survival of serous high-grade ovarian cancer displayed for two quantiles (5%,95%) based on Cox regression for TCGA-RNASeq/V2 data set. Numbers mean LATS expression for each quantile

A previous study has reported that LATS protein expression was associated with better outcome in a Chinese ovarian cancer cohort (Xu et al. 2015). Here, we analyzed *LATS1* and *LATS2* gene expression in publicly available ovarian cancer transcriptomic data sets. The Forest plot presentation over all data sets showed that high *LATS1* expression (Fig. 1a) was not associated with lower survival in ovarian cancer patients (HR = 1.00; *p* < 0.9176; 15 data sets). The results indicated inter-data set heterogeneity: Among these data sets, four showed an HR > 1.10 and three an HR < 0.90, and the TCGA and the TCGA RNASeqV2 displayed opposing HR values (0.94 vs 1.14). In contrast, high *LATS2* expression (Fig. 1b) was associated with a significant 19% higher risk for poorer survival (HR = 1.19; *p* < 0.0001; 10 data sets). This association for *LATS2* was found in eight out of nine data sets (HR ≥ 1.07), whereas an inverse association was found only in the GSE18520 data set (HR = 0.85). Interestingly, TCGA RNASeqV2 data for serous high-grade ovarian cancer showed that both *LATS1* and *LATS2* expressions were associated with a higher risk for poorer survival (HR = 1.14 for *LATS1* and HR = 1.08 for *LATS2*) for this more homogenous group of patients. This unfavorable outcome was confirmed in Kaplan–Meier curves, where log(*LATS1*) and log(*LATS2*) were computed on survival for two quantile (5%,95%) using the Cox regression model (Fig. 2a, b).

Collectively, neither *LATS1* nor *LATS2* expression was associated with favorable survival in ovarian cancer and *LATS2* expression may even have a negative effect on survival.

### LATS protein expression is not associated with better outcome in ovarian cancer (Swiss cohort)

Transcriptomic data analysis on LATS in ovarian cancer patients showed results discrepant from those of the Chinese cohort (Xu et al. 2015). We therefore immunohistochemically analyzed LATS expression on TMAs from a Swiss ovarian cancer cohort and compared LATS1 expression to tumor type, histology, staging, grading, and survival. The overall cohort consisted of 196 patients with ovarian tumors including benign cystadenomas, borderline tumors, and carcinomas, with the following clinicopathological characteristics (Table 1).

**Fig. 3:**
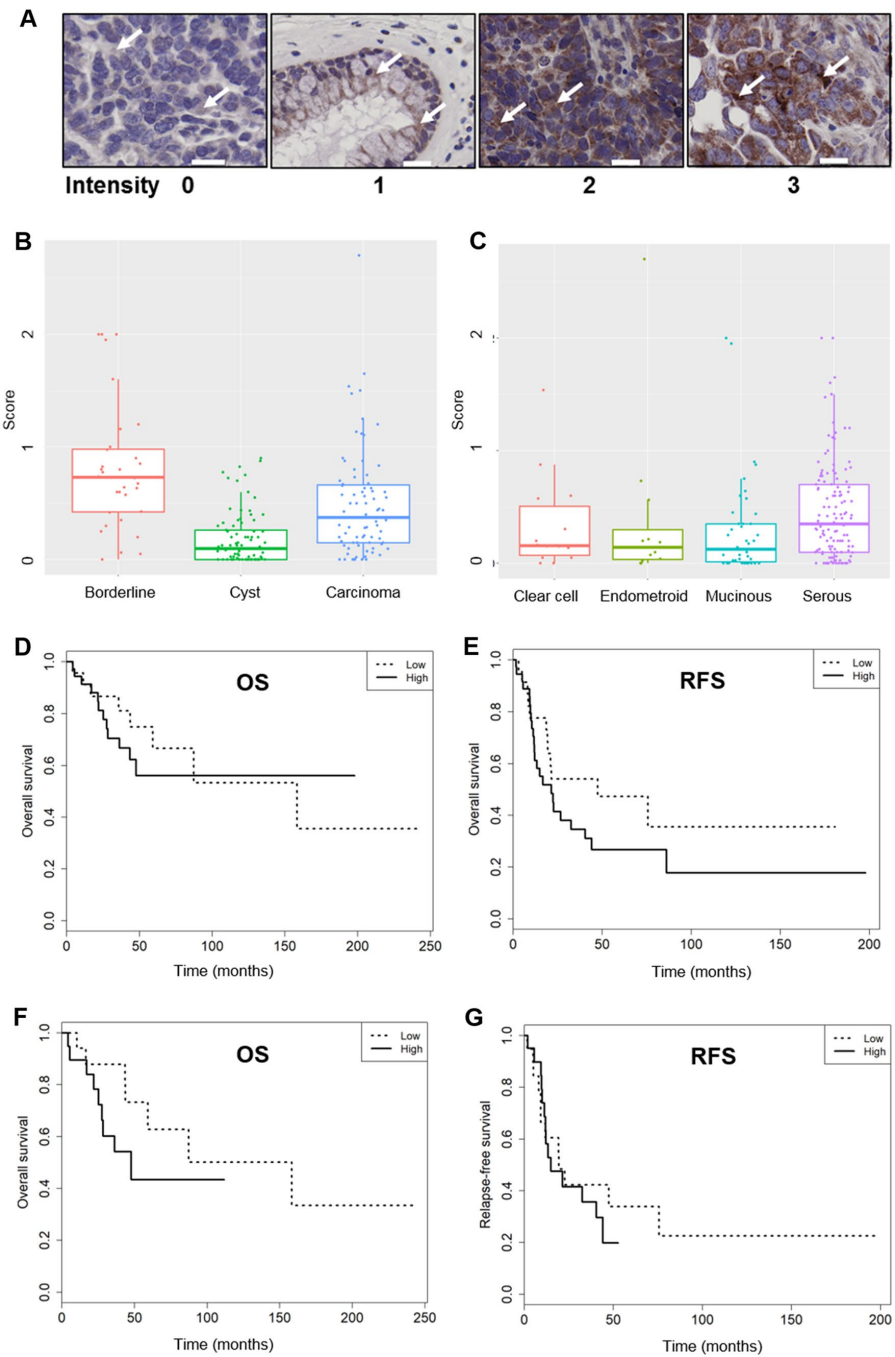
LATS1/2 protein expression related to cancer type, histological subtypes, and survival in the overall cohort and to survival in the serous carcinoma sub-cohort. **a** Representative example for LATS1/2 staining intensity from 0 to 3 (arrows). Scale bar indicates 20 μm. **b** LATS1/2 expression in cystadenoma, borderline, and carcinoma patients in the overall cohort (*n* = 196). Brackets indicate statistical significance, *p* < 0.05. **c** LATS1/2 expression in clear cell, endometrioid, mucinous, and serous histotypes in the overall cohort (*n* = 196). Brackets indicate statistical significance, *p* < 0.05. **d**, **e** Kaplan–Meier curves for low (dotted line) and high (straight line) LATS1/2 expression for OS (median months: 158.3 vs NE; *p* = 0.603) and for RFS (median months: 47.4 vs 21.4; *p* = 0.152) in the overall cohort. **f**, **g** Kaplan–Meier curves for low (dotted line) and high (straight line) LATS1/2 expression for OS (median months: 158.3 vs 47.6; *p* = 0.164) and for RFS (median months: 19.4 vs 14.9; *p* = 0.576) for the serous carcinoma sub-cohort

A representative example for LATS1/2 staining intensities (from 0 to 3) is shown in Fig. 3a. LATS1/2 protein expression was significantly different (*p* < 0.001) among the tumor types: it was lowest in cystadenoma, intermediate in carcinoma, and highest in borderline (Table 2, Fig. 3b). LATS1/2 protein expression was significantly higher in the serous than in the mucinous (*p* = 0.005) subtype, whereas it was similar for all other comparisons (Table 2, Fig. 3c). LATS1/2 protein expression was similar for FIGO stage I + II and stage III + IV in borderline tumors and carcinomas and was also similar in grade 1 compared to grade 2 and grade 3 carcinomas (Table 2). LATS1/2 protein expression was then related to outcome. The Kaplan–Meier curves showed that overall survival (OS) and relapse-free survival (RFS) were, despite an apparent 2.2-fold advantage of low LATS1/2-expressing patients for RFS (median: 47.4 months vs 21.4 months), not significantly different (*p* = 0.603 and *p* = 0.152, respectively) (Table 3, Fig. 3d, e). Taken together, LATS1/2 protein expression was higher in carcinomas than in cystadenomas, was higher in serous than in mucinous cancers, and was FIGO stage independent.

**Table 2:** Scores (median, IQR) and comparisons (overall cohort)

	Median	IQR	*N*	*p* global	*p* individual
Tumor type			196	< 0.001*	
Cystadenoma	0.100	0.263	85		
Borderline	0.731	0.558	32		
Carcinoma	0.375	0.513	79		
Borderline vs cystadenoma					**< 0.001**
Borderline vs carcinoma					**0.004**
Cystadenoma vs carcinoma					**< 0.001**
Histological subtype			196	**0.024**	
Clear cell	0.156	0.436	14		
Endometrioid	0.144	0.265	12		
Mucinous	0.125	0.338	43		
Serous	0.350	0.600	127		
Clear cell vs endometrioid					0.676
Clear cell vs mucinous					0.529
Clear cell vs serous					0.287
Endometrioid vs mucinous					0.929
Endometrioid vs serous					0.124
Mucinous vs serous					**0.005**
Stage			107	0.709	
I + II	0.397	0.703	48		
III + IV	0.500	0.522	59		
(I + II) vs (III + IV)					0.709
Grade			110	**0.002**	
1	0.239	0.572	4		
2^a^	0.233	0.519	23		
3	0.500	0.550	51		
Borderline	0.731	0.558	32		
1 vs 2					0.858
1 vs 3					0.432
2 vs 3					**0.044**
Borderline vs 1					0.085
Borderline vs 2					**< 0.001**
Borderline vs 3					**0.025**

*p < 0.05 = significant

*IQR* interquartile range, *N* number of patients

^a^Includes the G2 serous high-grade carcinomas

**Table 3:** Outcome statistics (overall cohort)

	Median	0.95 LCL; 0.95 UCL^a^	*N*	*p* value*
Overall cohort				
Overall survival			50	0.603
Median months (low)	158.3	59.1; NE		
Median months (high)	NE	43.4; NE		
Relapse-free survival				
Median months (low)	47.4	19.4; NE	50	0.152
Median months (high)	21.37	11.8; 44.1		
Serous sub-cohort				
Overall survival			39	
Median months (low)	158.3	59.1; NE		0.165
Median months (high)	47.7	27.5; NE		
Relapse-free survival			39	
Median months (low)	19.4	9.2; NE		0.576
Median months (high)	14.9	11.8; NE		

*N* number of patients, *NE* not estimable

*p < 0.05 = significant

^a^Lower; upper confidential level

Specifically interested in the serous ovarian carcinoma, we then analyzed LATS1/2 expression in this group of patients, which was also the largest histological subtype (*n* = 52) in our cohort. The descriptive statistics are summarized in Table 4. Among these 52 patients, a good three-quarters (77%) were FIGO stage I + II and just under one-quarter (23%) were FIGO stage III + IV. All except one among all the 51 were high-grade serous carcinoma patients. LATS1/2 protein expression in FIGO stage I + II and stage III + IV in this sub-cohort was not significantly different (*p* = 0.275) (Table 5). LATS12/2 expression in the one low-grade patient was higher than in the high-grade serous carcinoma patients (Table 5), but the meaning of this comparison is limited (one single case in the low-grade group). Both overall survival and relapse-free survival were not different in low LATS1/2- and high LATS1/2-expressing patients (*p* = 0.164 and *p* = 0.576, respectively) (Table 5, Fig. 3f, g); however, an at least apparent advantage of low LATS1/2-expressing serous carcinoma patients for overall survival was noted: the median overall survival of high LATS1/2 expressers was shorter than that of low LATS1/2 expressers. LATS1/2 expression was thus FIGO stage independent and did not associate with outcome. Taken together, the results from our Swiss cohort indicate that LATS1/2 expression was not associated with better outcome.

**Table 4:** Serous carcinoma cohort description

Adenocarcinoma		***N***
		52
Stage (FIGO)		48
I + II	11 (22.9%)	
III + IV	37 (77.1%)	
Grade		51
Low^a^	1 (2%)	
High^a^	50 (98%)	
Score^b^	0.50 [0.25;0.71]	52
Medsplit^c^		52
Low expression	28 (53.8%)	
High expression	24 (46.2%)	

*N* number of patients

^a^Low = G1; high = (G2 + G3); ^b^median and IQR (interquartile range); ^c^median split

**Table 5:** Scores (median, IQR) and comparisons (serous carcinoma cohort)

	Median	IQR	*N*	*p* value
Stage			48	
I + II	0.338	0.377	11	0.275
III + IV	0.550	0.494	37	
(I + II) vs (III + IV)				0.275
Grade			51	
Low	1.117	NE	1	0.081
High	0.500	0.456	50	

*IQR* interquartile range, *N* number of patients, *NE* not estimable

### LATS protein expression in ovarian cancer cell lines is not associated with drug sensitivity and EMT status

**Fig. 4:**
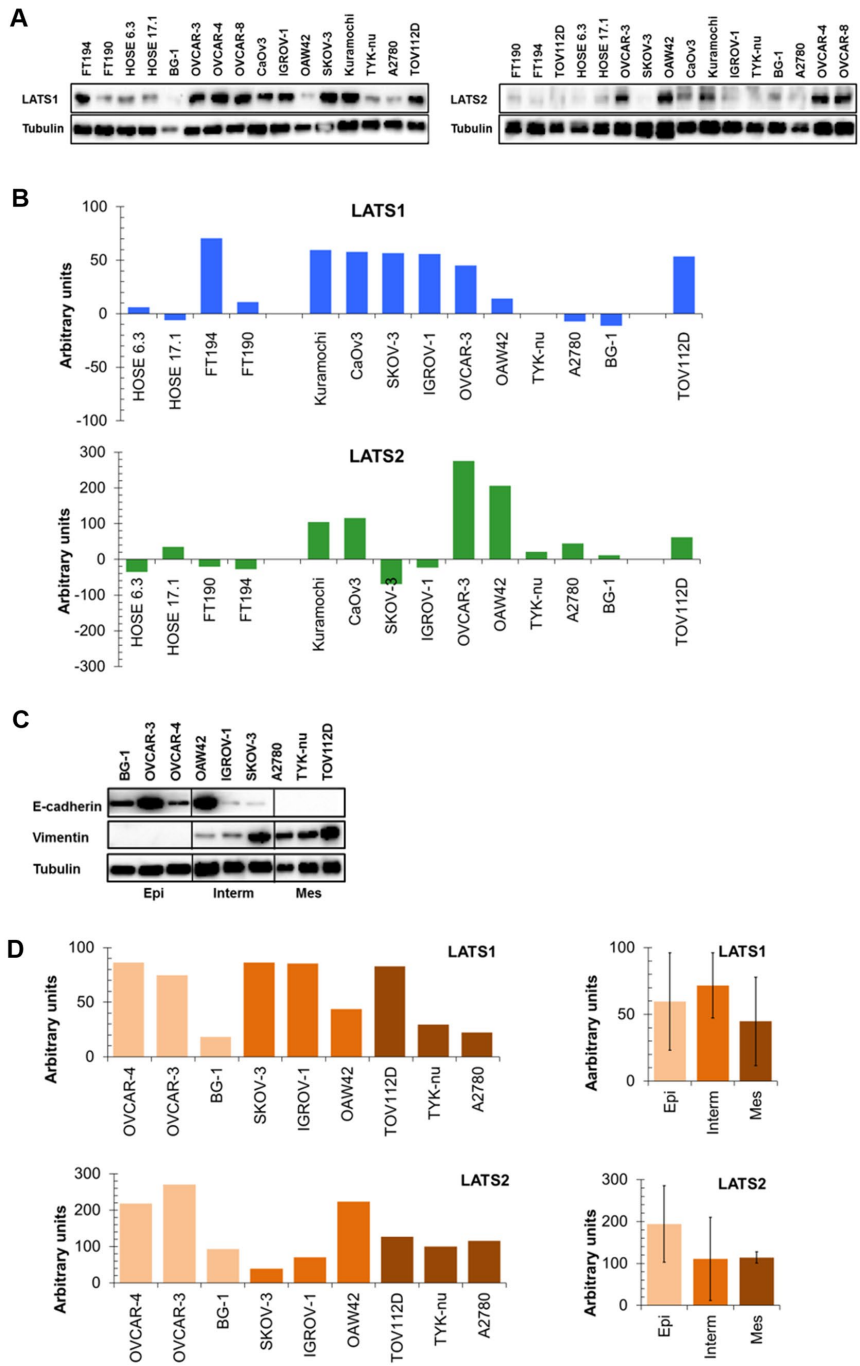
LATS1 and LATS2 protein expression, EMT status, and drug sensitivity in ovarian cancer cell lines. Expression of LATS1 and LATS2 in human ovary surface epithelial (HOSE 6.3 and HOSE 17.1), in fallopian tube (FT194 and FT190), in serous ovarian cancer (SOC: Kuramocchi, CaOv3, SKOV-3, IGROV-1, OVCAR-3, OVCAR-4, OVCAR-8, OAW42, TYK-nu, A2780, and BG-1), endometrioid ovarian cancer (EnOC: TOV112D) cell lines as determined by Western analysis (**a**: representative examples) and quantified by densitometry (**b**: presented as arbitrary units relative to LATS1 and LATS2 expression normalized for the average of the two HOSE cell lines). The cell lines were sorted by declining LATS1 expression (upper panel) and the same order was kept for LATS2 expression (lower panel). Densitometry data: mean of at least four independent experiments. **c** E-cadherin and vimentin expression: the cell lines were classified into “epithelial” (E-cadherin positive/vimentin negative), “intermediate” (E-cadherin positive/vimentin positive), and “mesenchymal” (E-cadherin negative/vimentin positive) according to (Jacob et al. 2018). **d** LATS1 and LATS2 expression (arbitrary units) in ovarian cancer cell lines classified as epithelial (light brown), intermediate (brown), and mesenchymal (dark brown). Left panel (LATS1 and LATS2 expression of all individual cell lines) and right panel (average of LATS1 and LATS2 expression in each class)

We were intrigued by the transcriptomic and our patient cohort immunohistochemistry data, suggesting that LATS expression was not favorable for outcome in ovarian cancer and was not less expressed in cancer tissue compared to the benign tumor tissue (cystadenomas). We therefore determined LATS protein expression by Western blotting in a panel of serous ovarian cancer (SOC), fallopian tube (FT), and human ovarian surface epithelial (HOSE) cell lines and wondered whether LATS protein expression was accordingly elevated in SOC cell lines compared to FT and HOSE cells. The results (Fig. 4a, b) demonstrate that in comparison with the HOSE cells, LATS1 was elevated in five out of nine SOC cell lines and in EnOC cells and TOV112D cells. FT194 cells displayed elevated LATS1, while the FT190 cells displayed LATS1 expression similar to the two HOSE cell lines. LATS2 expression was elevated in four out of nine SOC cell lines, while the five other SOC cell lines and the EnOC cell line displayed LATS expression comparable to HOSE cells. Both FT cell lines displayed LATS2 expression comparable to HOSE cells. Interestingly, Kuramocchi, CaOv3, and OVCAR-3 SOC cells had elevated LATS1 and LATS2, whereas A2780, TYK-nu, and BG-1 had LATS1 and LATS2 levels comparable to HOSE cells. SKOV-3, IGROV-1, and OAW42 cells had elevated LATS1 but low LATS2, indicating that in six out of nine SOC cell lines LATS1 and LATS2 levels were comparable. These data indicate that LATS1 and LATS2 are elevated in at least some SOC cell lines in comparison to the HOSE cells.

We then determined whether LATS protein expression was associated with the EMT status of SOC cells, e.g., whether LATS protein expression decreased with EMT. The cell lines were classified according to their expression of E-cadherin (epithelial marker) and vimentin (mesenchymal marker) as epithelial (E-cad +/vim−), mesenchymal (E-cad−/vim +), and intermediate (E-cad +/vim +) **(**Fig. 4c; Jacob  et al. 2018). LATS1 and LATS2 expression generally varied within each individual EMT class, but was largely comparable among the three EMT classes **(**Fig. 4d), indicating that LATS expression did not indicate the EMT status of the cells.

We next determined whether LATS protein expression was an indicator for drug sensitivity, i.e., whether ovarian cancer cell lines with higher LATS were drug sensitive and those with low LATS were drug resistant. Spearman’s rank correlation showed that there was no statistically significant association between LATS expression and sensitivity to cisplatin, carboplatin, doxorubicin, and paclitaxel (Table 6). This indicates that LATS protein expression was not associated with the extent of drug response.

**Table 6:** LATS expression and drug sensitivity correlation in ovarian cancer cell lines

Drug	LATS1		LATS2	
	Correlation*	*p* value*	Correlation	*p* value
Cisplatin	0.571	0.151	− 0.381	0.360
Carboplatin	0.619	0.115	− 0.381	0.360
Doxorubicin	0.571	0.151	− 0.214	0.619
Paclitaxel	0.619	0.115	− 0.476	0.234

*Spearman’s rank correlation

**Fig. 5:**
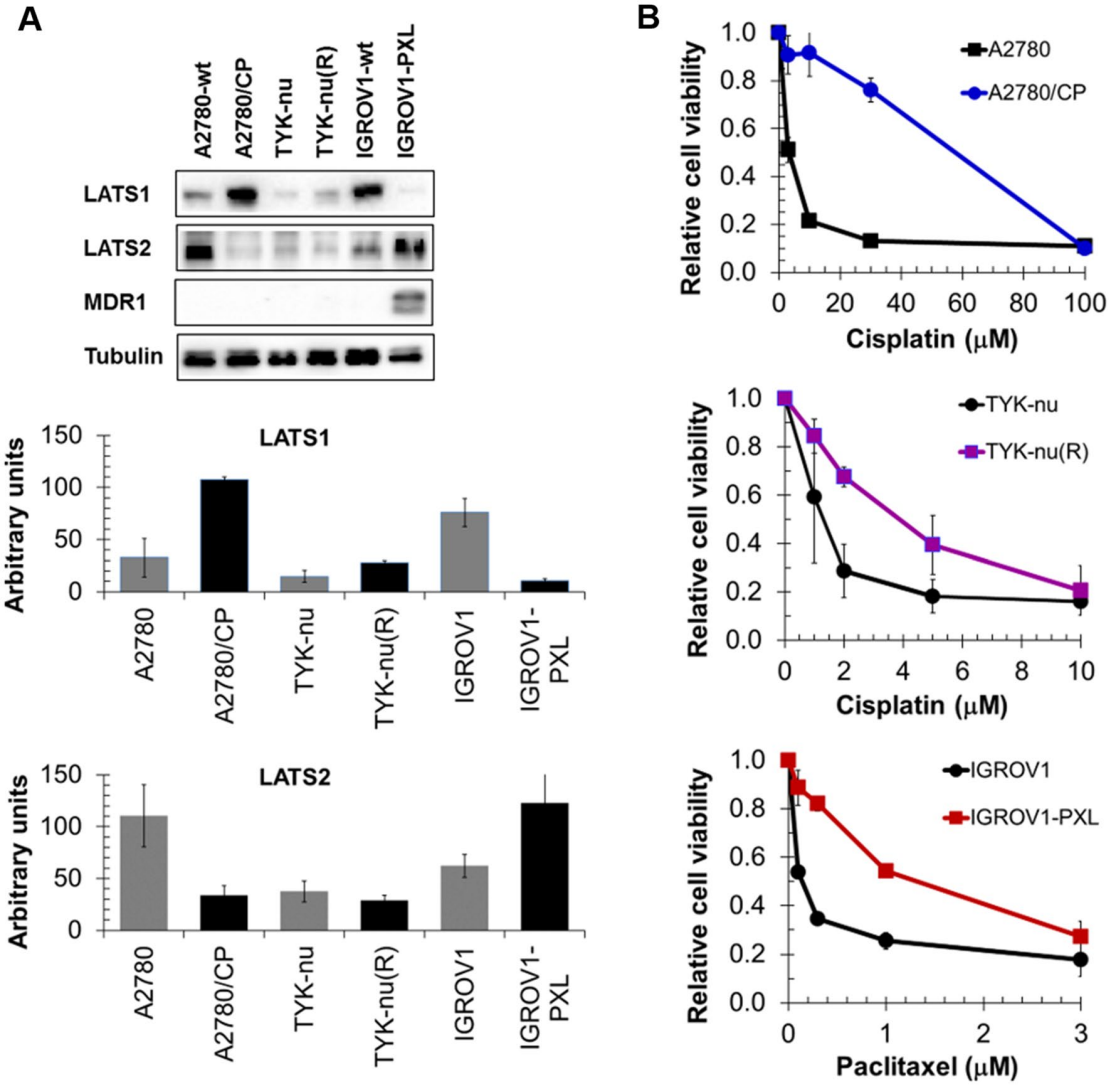
LATS1, LATS2, and MDR1 expression (**a**) in cisplatin-resistant A2780/CP (16-fold) and TYK-nu(R) (4-fold: Kohler et al. 2017) and paclitaxel-resistant IGROV1-PXL (9.3-fold; Kohler et al. 2017) cells and in the respective parental cell lines. Resistance determined for IC50 values from MTT assays (**b**)

We also wondered whether low LATS-expressing cells that acquired resistance through multiple exposures to increasing drug concentrations displayed elevated LATS expression. The results demonstrate that acquired cisplatin resistance in A2780/CP cells and in TYK-nu(R) cells (to a lesser extent though) was associated with elevated LATS1, but that inversely acquired paclitaxel resistance in IGROV1-PXL cells was associated with reduced LATS1 (Fig. 5a, b). In contrast, LATS2 was decreased in these cisplatin-resistant A2780/CP cells, whereas it was comparably expressed in cisplatin-resistant TYK-nu(R) cells, and even markedly elevated in the paclitaxel-resistant IGROV1-PXL cells. Acquired paclitaxel resistance in IGROV1-PXL cells associated with de novo expression of multidrug resistance protein MDR1. Collectively, LATS protein expression levels did not generally indicate the extent of drug sensitivity.

## Discussion

We investigated LATS expression in publicly accessible transcriptomic data sets and in tissue samples from a Swiss ovarian cancer patient cohort with regard to clinicopathological characteristics and outcome and based on the intriguing results then determined in a panel of ovarian cancer cell lines whether LATS expression is an indicator for the EMT status of these cells and for drug sensitivity. Based on the results, we may conclude that LATS expression (a) did not associate with better outcome in ovarian cancer, (b) was not reduced in SOC cell lines, and (c) was not associated with EMT status and drug sensitivity.

Several interesting findings emerged from this study: Firstly, the analysis of 15 databases indicated that neither *LATS1* nor *LATS2* expression was associated with favorable survival in ovarian cancer and that *LATS2* may even have a negative role for survival. They neither support the putative function of LATS proteins as tumor suppressors nor are they consistent with the previous study of ovarian cancer patients, where elevated LATS1 and LATS2 expression was associated with better outcome in serous ovarian cancer patients (Xu et al. 2015). Rather, *LATS2* may function as a possible tumor promoter. However, it is worthwhile mentioning that the interpretation of the available transcriptomic data may be done with care because whether an association or its reverse was present depended on the data set selected for the analysis.

Secondly, LATS1/2 expression was not favorable for overall survival in both the overall cohort and the serous carcinoma sub-cohort. It even tended to have a negative effect on relapse-free survival, but the informative value is limited by the low number of events. These results are inconsistent with a tumor-suppressive function of LATS family proteins, but support the findings from the transcriptomic data sets analysis. Histologically, LATS1/2 expression was significantly higher in the serous than in the mucinous subtype, whereas it was comparable among the endometrioid, clear cell, and mucinous subtypes. LATS1/2 expression was lowest in cystadenoma, intermediate in carcinoma, and highest in borderline. LATS1/2 expression was not different among the different FIGO stages for both the overall tumor patient cohort and the serous carcinoma sub-cohort. Of note, LATS1/2 expression was significantly higher in grade 3 than grade 2 in the overall cohort: a statement not meaningful for the serous carcinoma sub-cohort, because only one single case was available in the low-grade group.

These results are discrepant to those from the previous study by Xu et al. who reported that LATS1 and LATS2 expression was elevated in mucinous compared to serous ovarian carcinomas, correlated with better outcome (longer survival and less recurrence), decreased with higher FIGO stage, and was elevated in high-grade serous carcinomas (Xu et al. 2015). The reasons for this discrepancy are unclear, but may be at least partly explained if LATS had a function different from that of a tumor suppressor. It is unlikely that the use of an antibody recognizing total LATS 1/2 protein instead of individual antibodies for LATS1 and LATS2 accounted for the discrepant immunohistochemistry results. On the other hand, our immunohistochemistry results are largely in line with those from transcriptomic data sets: those did not display an unfavorable outcome for ovarian cancer patients with low *LATS* expression as expected if LATS was tumor suppressive. Our data are, however, consistent with a previous study reporting LATS overexpression in nasopharyngeal cancer (Zhang et al. 2010).

Thirdly, we also report on issues not yet addressed in ovarian cancer, such as the possible relationship between LATS protein expression in ovarian cancer cell lines and their EMT status and their drug sensitivity. Interestingly LATS1 and LATS2 proteins were higher or at least comparably expressed in ovarian cancer cell lines compared to “normal” HOSE and FT cell lines, but in no case LATS protein expression was decreased relative to the “normal” cell lines. These results may be surprising: assuming a tumor-suppressive function of LATS and considering the decreased LATS expression in SOC compared to healthy tissue reported previously (Xu et al. 2015), LATS protein expression would have been expected to be lower in HOSE and FT cells than in SOC cells. On the other hand, they seem consistent with the transcriptomic data set results. Also, expression of LATS1 and LATS2 is not necessarily interdependent, meaning that high LATS1 expression does not necessarily mean high LATS2 expression in ovarian cancer cell lines. Taken together, the results do not support the proposed function of LATS proteins as tumor suppressors.

Likewise, LATS1 and LATS2 expression was comparable among epithelial, intermediate, and mesenchymal ovarian cancer cell lines, indicating that LATS protein expression was not associated with the cells’ EMT status. This is at odds with the view that LATS expression declines when cells transition from epithelial to mesenchymal (Nozaki et al. 2019; Moroishi et al. 2015; Lei et al. 2008). Opposed to these studies is a report showing that LATS can potentiate tumor-promoting factors and augment EMT (Zhang et al. 2012).

In addition, the expression of LATS1 and LATS2 was not associated with drug sensitivity, meaning that the extent of LATS expression in SOC cell lines did in general not indicate whether cells responded better or poorer to chemotherapeutic drug exposure. However, it may do so in individual cases: although lacking statistical significance, cells with higher LATS1 tended to be drug resistant and those with higher LATS2 tended to be drug sensitive, suggesting that LATS1 and LATS2 have opposing functions in this context. Notably, the absence of an association between LATS1 and LATS2 expression and drug sensitivity does not imply that LATS proteins are not implicated. Indeed, LATS1/2 does modulate cellular responses to chemotherapeutic drug exposure. Low *LATS* expression improved therapy response in advanced and recurrent breast cancer patients, possibly through the LATS downregulation-induced disruption of cell cycle checkpoints (Takahashi et al. 2007). Conversely, loss of LATS1 rendered HeLa cells resistant to paclitaxel-induced cell death (Yeung et al. 2018) and loss of LATS2 resulted in doxorubicin and etoposide resistance in leukemic cell lines (Kawahara et al. 2008). The molecular mechanisms by which LATS governs drug sensitivity or resistance are, however, not understood. We also wondered whether acquired drug resistance involved alterations in LATS protein expression. Indeed, altered LATS protein expression was found, but an obvious pattern was missing: this means that acquired resistance was not generally associated with elevated LATS1 and LATS2. Whether the de novo expression of multidrug resistance transporter MDR1 and the altered LATS expression in the paclitaxel-resistant IGROV1-PXL cells are connected cells is unknown.

In summary, our results suggest a function of LATS proteins different from that of a tumor suppressor and may even point to an opposed function of LATS proteins in ovarian cancer. The role of LATS proteins hence remains controversial and possibly is context dependent and cancer type dependent.

## References

[CR1] Bowtell DD, Böhm S, Ahmed AA, Aspuria PJ, Bast RC, Beral V, Berek JS, Birrer MJ, Blagden S, Bookman MA (2015). Rethinking ovarian cancer II: reducing mortality from high-grade serous ovarian cancer. Nat Rev Cancer.

[CR2] Furth N, Aylon Y (2017). The LATS1 and LATS2 tumor suppressors: beyond the Hippo pathway. Cell Death Differ.

[CR3] Ganzfried BF, Riester M, Haibe-Kains B, Risch T, Tyekucheva S, Jazic I, Wang XV, Ahmadifar M, Birrer MJ, Parmigiani G (2013). CuratedOvarianData: clinically annotated data for the ovarian cancer transcriptome. Database (Oxford).

[CR4] Jacob F, Ukegjini K, Nixdorf S, Ford CE, Olivier J, Caduff R, Scurry JP, Guertler R, Hornung D, Mueller R (2012). Loss of secreted frizzled-related protein 4 correlates with an aggressive phenotype and predicts poor outcome in ovarian cancer patients. PLoS One.

[CR5] Jacob F, Alam S, Konantz M, Liang CY, Kohler RS, Everest-Dass AV, Huang YL, Rimmer N, Fedier A, Schötzau A (2018). Transition of mesenchymal and epithelial cancer cells depends on α1–4 galactosyltransferase-mediated glycosphingolipids. Cancer Res.

[CR6] Janse van Rensburg HJ, Yang X (2016). The roles of the Hippo pathway in cancer metastasis. Cell Signal.

[CR7] Jiménez-Velasco A, Román-Gómez J, Agirre X, Barrios M, Navarro G, Vázquez I, Prósper F, Torres A, Heiniger A (2005). Downregulation of the large tumor suppressor 2 (LATS2/KPM) gene is associated with poor prognosis in acute lymphoblastic leukemia. Leukemia.

[CR8] Kawahara M, Hori T, Chonabayashi K, Oka T, Sudol M, Uchiyama T (2008). Kpm/Lats2 is linked to chemosensitivity of leukemic cells through the stabilization of p73. Blood.

[CR9] Kohler RS, Kettelhack H, Knipprath-Mészaros AM, Fedier A, Schoetzau A, Jacob F, Heinzelmann-Schwarz V (2017). MELK expression in ovarian cancer correlates with poor outcome and its inhibition by OTSSP167 abrogates proliferation and viability of ovarian cancer cells. Gynecol Oncol.

[CR10] Lei QY, Zhang H, Zhao B, Zha ZY, Bai F, Pei XH, Zhao S, Xiong Y, Guan KL (2008). TAZ promotes cell proliferation and epithelial-mesenchymal transition and is inhibited by the hippo pathway. Mol Cell Biol.

[CR11] Lin XY, Zhang XP, Wu JH, Qiu XS, Wang EH (2014). Expression of LATS1 contributes to good prognosis and can negatively regulate YAP oncoprotein in non-small-cell lung cancer. Tumour Biol.

[CR12] Masuda H, Ozols RF, Lai GM, Fojo A, Rothenberg M, Hamilton TC (1988). Increased DNA repair as a mechanism of acquired resistance to cis-diamminedichloroplatinum (II) in human ovarian cancer cell lines. Cancer Res.

[CR13] Meng Z, Moroishi T, Guan KL (2016). Mechanisms of Hippo pathway regulation. Genes Dev.

[CR14] Morinaga N, Shitara Y, Yanagita Y, Koida T, Kimura M, Asao T, Kimijima I, Takenoshita S, Hirota T, Saya H (2000). Molecular analysis of the h-warts/LATS1 gene in human breast cancer. Int J Oncol.

[CR15] Moroishi T, Hansen CG, Guan KL (2015). The emerging roles of YAP and TAZ in cancer. Nat Rev Cancer.

[CR16] Nozaki M, Yabuta N, Fukuzawa M, Mukai S, Okamoto A, Sasakura T, Fukushima K, Naito Y, Longmore GD, Nojima H (2019). LATS1/2 kinases trigger self-renewal of cancer stem cells in aggressive oral cancer. Oncotarget.

[CR17] Ozols RF (2006). Challenges for chemotherapy in ovarian cancer. Ann Oncol.

[CR18] Plouffe SW, Hong AW, Guan KL (2015). Disease implications of the Hippo/YAP pathway. Trends Mol Med.

[CR19] Son MW, Song GJ, Jang SH, Hong SA, Oh MH, Lee JH, Baek MJ, Lee MS (2017). Clinicopathological significance of large tumor suppressor (LATS) expression in gastric cancer. Gastric Cancer.

[CR20] Takahashi Y, Miyoshi Y, Takahata C, Irahara N, Taguchi T, Tamaki Y, Noguchi S (2005). Down-regulation of LATS1 and LATS2 mRNA expression by promoter hypermethylation and its association with biologically aggressive phenotype in human breast cancers. Clin Cancer Res.

[CR21] Takahashi Y, Miyoshi Y, Morimoto K, Taguchi T, Tamaki Y, Noguchi S (2007). Low LATS2 mRNA level can predict favorable response to epirubicin plus cyclophosphamide, but not to docetaxel, in breast cancers. J Cancer Res Clin Oncol.

[CR22] Visser S, Yang X (2010). LATS tumor suppressor: a new governor of cellular homeostasis. Cell Cycle.

[CR23] Xu B, Sun D, Wang Z, Weng H, Wu D, Zhang X, Zhou Y, Hu W (2015). Expression of LATS family proteins in ovarian tumors and its significance. Hum Pathol.

[CR24] Yeung B, Khanal P, Mehta V, Trinkle-Mulcahy L, Yang X (2018). Identification of Cdk1-LATS-Pin1 as a novel signaling axis in anti-tubulin drug response of cancer cells. Mol Cancer Res.

[CR25] Yoshiya N, Adachi S, Misawa Y, Yuzawa H, Honda T, Kanazawa K, Takeuchi S, Tanaka K (1989). Isolation of cisplatin-resistant subline from human ovarian cancer cell line and analysis of its cell-biological characteristics. Nihon Sanka Fujinka Gakkai Zasshi.

[CR26] Yu FX, Zhao B, Guan KL (2015). Hippo pathway in organ size control, tissue homeostasis, and cancer. Cell.

[CR27] Zanconato F, Cordenonsi M, Piccolo S (2016). YAP/TAZ at the roots of cancer. Cancer Cell.

[CR28] Zhang Y, Hu CF, Chen J, Yan LX, Zeng YX, Shao JY (2010). LATS2 is de-methylated and overexpressed in nasopharyngeal carcinoma and predicts poor prognosis. BMC Cancer.

[CR29] Zhang K, Rodriguez-Aznar E, Yabuta N, Owen RJ, Mingot JM, Nojima H, Nieto MA, Longmore GD (2012). Lats2 kinase potentiates Snail1 activity by promoting nuclear retention upon phosphorylation. EMBO J.

